# Late Fracture of Stylet-driven Lead Intended for Conduction System Pacing 2 Years After Implant

**DOI:** 10.19102/icrm.2026.17052

**Published:** 2026-05-15

**Authors:** Francis J. Ha, Sing Huey Cheng, Jagat Adhikari, Adam Brown, Colin Machado

**Affiliations:** 1 Victorian Heart Institute and Victorian Heart Hospital, Monash University, Monash Health, Clayton, Australia

**Keywords:** Complications, conduction system pacing, lead fracture, left bundle branch area pacing, stylet-driven leads

## Abstract

Left bundle branch area pacing (LBBAP) has become adopted in recent years to achieve improved ventricular synchrony and reduce the risk of pacing-induced cardiomyopathy. It is a potential alternative to standard right ventricular pacing. However, long-term clinical data regarding lead integrity and longevity are still lacking. Several case reports have highlighted the risk of distal conductor lead failure. In this case, we report late distal lead conductor failure with the Solia S60 lead (Biotronik, Berlin, Germany) intended for left bundle branch area two years after initial implant.

## Introduction

Left bundle branch area (LBBA) pacing (LBBAP) has been adopted in recent years to achieve improved ventricular synchrony and reduce the risk of pacing-induced cardiomyopathy.^1^ It is a potential alternative to standard right ventricular (RV) pacing. However, long-term clinical data regarding lead integrity and longevity are still lacking. Several case reports have highlighted the risk of distal conductor lead failure up to 13 months.^2,3^ In this case report, we describe late distal lead conductor failure with the Solia S60 lead (Biotronik, Berlin, Germany) intended for LBBAP 2 years after the initial implant.

## Case presentation

A 79-year-old woman was referred for a single-chamber permanent pacemaker implant due to symptomatic slow atrial fibrillation (AF) with right bundle branch block **(Figure 1)**. Her symptoms were lethargy and shortness of breath on exertion, with a daytime resting heart rate of approximately 50–60 bpm. Her medical history was significant for hypertension, type 2 diabetes mellitus, and persistent AF. Her regular medications included warfarin, 32 mg of candesartan daily, 20 mg of atorvastatin daily, 40 mg of frusemide daily, 2.5 mg of indapamide daily, 500 mg of metformin twice daily, and 80 mg of gliclazide twice daily. She was living independently at home with the help of a single-point (walking) stick and was a non-smoker. Her preoperative transthoracic echocardiogram demonstrated normal left ventricular systolic function with mild functional mitral regurgitation and severe bi-atrial enlargement.

**Figure 1:**
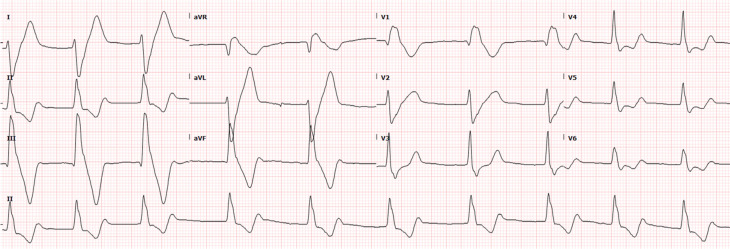
Electrocardiogram of baseline rhythm showing atrial fibrillation with right bundle branch block and slow ventricular rates.

In view of her longstanding AF with slow ventricular rates and anticipated high ventricular pacing burden, a decision was made to attempt conduction system pacing (CSP) in the first instance. The procedure was performed under light sedation and access was obtained through the axillary vein after a left-sided deltopectoral incision. A temporary pacing lead was inserted into the right ventricle for back-up pacing support. A Selectra 3D sheath (55-mm curve, 42 cm long; Biotronik) was used to deploy a Biotronik Solia S60 active fixation lead into the LBBA. True LBBA capture could not be obtained despite multiple attempts with the lead according to conventional LBBAP parameters.^4^ Finally, a deep mid-septal position was accepted, with a paced QRS duration of 142 ms **(Videos 1 and 2 and Figure 2)**. The lead was connected to a single-chamber Biotronik Evity 8 SR-T device. Her lead parameters at implant showed a threshold of 0.7 V at 0.4 ms pulse width in bipolar polarity, R-wave sensing at 9 mV, and an impedance of 546 Ω. She was programmed to VVI 60, and the output was set to a 1.3 V at 0.4 ms pulse width. Her lead parameters were stable during the day 1 post-implant check with 98% ventricular pacing burden. She was followed up externally by her referring cardiologist after implant with regular device checks.

**Figure 2:**
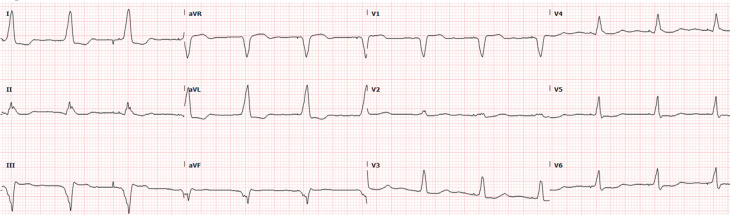
Electrocardiogram of paced morphology after lead implantation.

Two years later, she presented to the emergency department after an unwitnessed fall with head strike. Computed tomography of the head and cervical spine showed a small-volume bilateral superior frontal lobe subarachnoid hemorrhage with no fractures. Her presenting rhythm was slow AF with a heart rate of 35 bpm **(Figure 3)**. A device check revealed a sudden change in lead parameters about 6 weeks prior, with a sharp rise in impedance (>3000 Ω), loss of R-wave sensing, and exit block at maximal output in unipolar and bipolar configurations **(Figure 4)**. Lead parameters prior to that point were stable. A chest X-ray demonstrated acute angulation of the lead tip compared with the initial chest X-ray performed on the day of implant **(Figure 5)**. Computed tomography of the chest and pulmonary arteries was initially performed in the emergency department for investigation of her syncope, which showed the lead in the interventricular septum with no pericardial effusion **(Video 3)**. Following a period of neurological observation for her intracranial hemorrhage with warfarin withheld, a consensus decision was made to briefly attempt lead extraction and insert a new RV lead. Fluoroscopic imaging confirmed abnormal angulation and conductor fracture between the ring and tip electrode **(Figure 6)**. An attempt to remove the original lead was made; however, the helix could not be retracted and the lead was left capped. A new Biotronik Solia S60 lead was inserted into a RV apical position for stability with acceptable lead parameters (threshold, 0.6 V at 0.4 ms in bipolar configuration; R-wave sensing, 7.1 mV; and impedance, 663 Ω), and she was discharged from hospital the next day.

**Figure 3:**
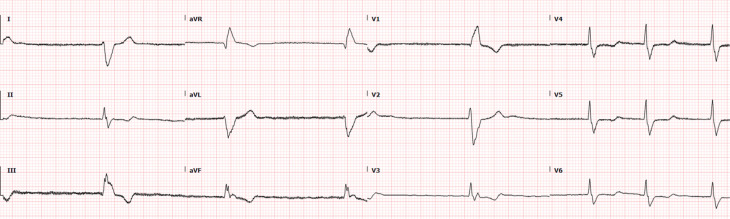
Electrocardiogram of slow AF with right bundle branch block despite pacemaker implantation.

**Figure 4:**
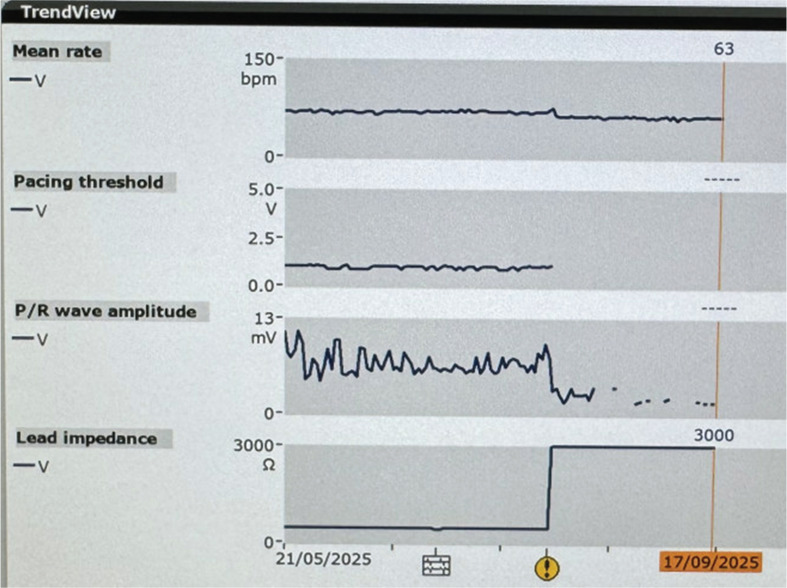
Device check demonstrating sudden change in lead parameters including ventricular pacing threshold, R-wave sensing, and lead impedance.

**Figure 5:**
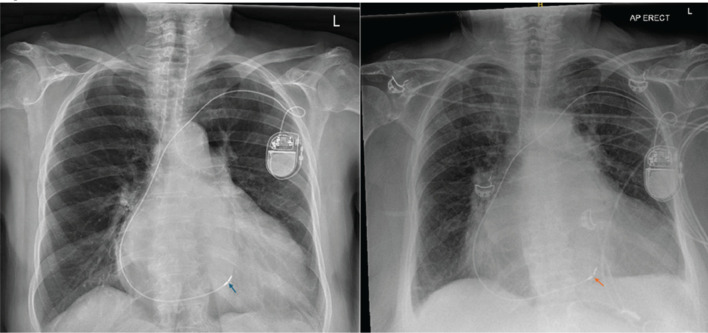
Initial chest X-ray after device implantation **(A)** and repeat chest X-ray at the time of syncope with fall 2 years later **(B)** demonstrating change in angulation of the distal lead between the ring electrode and lead tip electrode. The blue arrow shows initial angulation, with the orange arrow showing more acute angulation.

**Figure 6:**
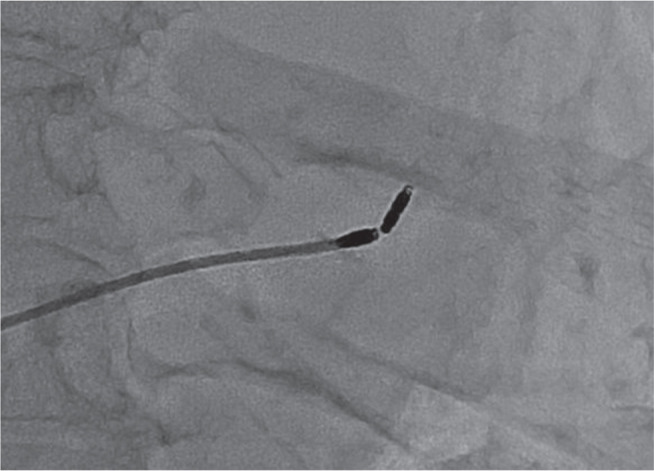
Fluoroscopic imaging showing probable fracture between the tip and ring electrode.

The patient verbally consented to the publication of her medical case in a peer-reviewed medical journal. The authors of this article, who actively participated in the decision process and management, obtained written informed consent from the patient, in accordance with Committee on Publication Ethics guidelines.

## Discussion

CSP, and specifically LBBAP, has become an alternative to conventional RV pacing to reduce the risk of pacing-induced cardiomyopathy.^1,5^ Numerous leads have been repurposed by device companies to be used in CSP, including both lumenless leads (LLLs) and stylet-driven leads (SDLs). The decision between an LLL and SDL is based on operator’s preference and experience. Potential advantages of LLLs include their smaller lead body and fixed helix design without concern for helix retraction, while SDLs benefit from enhanced stiffness and torque transmission with a larger-diameter delivery sheath providing better support.^6^ While both types of leads have been approved for standard RV pacing, their performance and longevity are still being established when deployed deeper into the interventricular septum for LBBA capture. During implant, comparative data from 925 consecutive patients undergoing LBBAP for bradycardia or cardiac resynchronization showed higher acute success with LLLs than with SDLs (95% vs. 85%), with SDLs tending to be implanted in a more inferior and mid-apical septal position.^7^ Additionally, acute lead-related complications were higher with SDLs than with LLLs (15.9% vs. 6.1%), although implant and fluoroscopy times were shorter with SDLs.^7,8^ A meta-analysis of observational studies nevertheless suggested no significant difference in the paced QRS duration between lead types.^9^

In relation to lead conductor failure, a study of 325 patients undergoing LBBAP reported that the incidence of early conductor failure with SDLs was 0.6% (2/325), which occurred between the tip housing and ring electrode, while no fractures occurred in standard RV pacing leads.^10^ Of note, high lead bending angulations were noted in 1.3% of LBBAP patients. Additional bench testing of excessive preconditioned leads showed a higher probability of early conductor fracture compared with standard preconditioned leads. In a multicenter observational study of 17 international centers enrolling 8255 patients with LBBAP, lead fracture rates were 0.04% in LLLs compared with 0.4% in SDLs at follow-ups of 19.5 and 10.3 months, respectively, which was a statistically significant difference.^11^

Both the stylet-driven Biotronik Solia S60 lead and Selectra 3D catheter system have Conformité Européenne (CE) and US Food and Drug Administration (FDA) approval for LBBAP.^12^ When used for LBBAP, a case series of three patients showed distal lead tip fractures at 4 weeks, 11 months, and 13 months after implantation.^3^ All demonstrated exit block during threshold testing with a bipolar impedance of >2500 Ω. Similarly, another case reported distal Biotronik Solia S60 lead conductor fracture 12 months after implant for LBBAP following vigorous exercise.^2^ Fluoroscopy suggested possible fracture at the hinge just before the ring electrode entry point into the septum. In our case, the abnormal angulation adjacent to the ring electrode is highly suspicious for distal lead tip fracture as well. Concerns have been raised about the implantation site and technique for LBBAP that could result in greater mechanical stress at the distal part of the lead with increased risk of fracture compared with conventional RV septal or apical positioning. This may be of greater relevance for SDLs, where presumably the presence of a lumen changes the tensile strength and potentially lead integrity when implanted deeper into the septum while leaving the ring electrode outside of the septum. Consideration should be given to deeper lead deployment into the septum to ensure the ring electrode is at least partially buried in the septum. This hopefully avoids the formation of a fulcrum point between the distal tip and ring electrode, which may be susceptible to increased risk of fracture with repetitive mechanical forces over time. However, this must be balanced with the risk of perforation through the left ventricular side of the septum. Late myocardial perforations beyond 1 month have also been reported with standard RV leads, and this risk is higher in cases with activation fixation leads.^13^ Conventional LBBAP parameters suggestive of perforation, such as negative current of injury during unipolar pacing, unipolar impedances of <500 Ω, or drops of >200 Ω, should be promptly recognized. Unipolar pacing with capture from the ring electrode can also be used to assess ring electrode contact with the interventricular septum. An additional consideration is the lead body diameter (1.8 mm/5.8 F) of the Biotronik Solia S60 lead, which is smaller than that of most other SDLs.^12^ It is unclear whether this design in combination with a lumen might contribute to an increased risk of fracture.^11^ It should be noted that, in this case, initial technical difficulties with lead deployment and repeated fixations could potentially affect the long-term lead integrity. However, the fracture did not appear to have occurred at the point of the helix. Furthermore, the patient’s enlarged right atrium also led to reduced slack in the lead and may have contributed to the risk of lead fracture.

## Conclusions

This case highlights the risk of late distal lead conductor fracture with the stylet-driven Biotronik Solia S60 lead when attempting LBBAP. To the best of our knowledge, this is the longest time period from implant to lead failure for this SDL in LBBAP reported in the literature thus far. Ongoing device surveillance for lead complications is needed for CSP given the present lack of long-term clinical data. Certain procedural factors, such as deeper deployment of the lead through the interventricular septum to avoid a fulcrum point occurring between the ring and tip electrode, can also be considered on a case-by-case basis.

## Supporting information

Supplementary Video 1: Left anterior oblique fluoroscopic view of Selectra 3D sheath with contrast injection to determine septum orientation.

Supplementary Video 2: Anterior-Posterior fluoroscopic view of implanted Biotronik Solia S60 in mid right ventricular septum.

Supplementary Video 3: Computed tomography of the chest and pulmonary arteries initially performed for investigation of syncope in the emergency department. The lead is implanted in the interventricular septum with no pericardial effusion.
